# Gut Microbiota Mediates Host Responses to Microplastic Exposure in *Artemia salina*

**DOI:** 10.3390/biology15100763

**Published:** 2026-05-11

**Authors:** Ruying Ma, Huiru Lu, Shisong Zhang, Hongli Ji, Fengjie Xin, Gang Wang

**Affiliations:** School of Life Science and Technology, Shandong Second Medical University, Weifang 261053, China; ryingma@126.com (R.M.); hruluk@126.com (H.L.); shisongz@126.com (S.Z.); hlijih@163.com (H.J.); 20240046@stu.sdsmu.edu.cn (F.X.)

**Keywords:** *Artemia salina*, polystyrene microplastics, microbial dysbiosis, host–microbe interaction, aquaculture, environmental toxicology

## Abstract

Background: Microplastics, derived from plastic degradation and industrial sources, are widely detected in aquatic environments and food systems, posing increasing environmental and ecological risks. Aims: This study aimed to investigate how microplastics affect host physiology and gut microbiota, as well as determine whether microbiota changes actively modulate host responses. Methods: Using *A. salina* as a model organism, we combined physiological assays, oxidative stress analysis, gut microbiome profiling, and bacterial functional validation under chronic polystyrene microplastics exposure. Results: Polystyrene microplastics accumulated in the gut and significantly impaired growth and survival, accompanied by reduced digestive enzyme activity and immune function, as well as increased oxidative stress, indicating disruption of physiological homeostasis. Microplastic exposure also induced microbial dysbiosis, characterized by decreased diversity and compositional shifts. Functional assays demonstrated that a bacterium enriched under exposure, *Pseudomonas knackmussii*, partially restored host growth and physiological functions while reducing oxidative stress. Conclusions: These findings demonstrate that gut microbiota are not only altered by microplastic exposure but also actively modulate host responses to environmental stress, providing new insight into microbiota-mediated resilience under pollutant stress.

## Simple Summary

Microplastics, derived from plastic degradation and industrial sources, are widely present in aquatic environments and food systems, posing increasing environmental risks highlighted by organizations such as WHO and FAO. However, how they affect host physiology and the role of gut microbiota remain unclear. Using the brine shrimp *Artemia salina* as a model, we show that microplastics accumulate in the gut and impair growth and survival. These effects are associated with reduced digestive enzyme activity, suppressed immune function, and increased oxidative stress, indicating disruption of physiological homeostasis. Microplastic exposure also reduces gut microbial diversity and alters community composition. Notably, *Pseudomonas knackmussii*, a bacterium enriched under exposure, partially restores host growth and physiological functions while alleviating oxidative stress. These findings demonstrate that gut microbiota can actively modulate host responses to environmental stress.

## 1. Introduction

Microplastic (MP) pollution has emerged as a pervasive and persistent threat to aquatic ecosystems due to its widespread distribution and resistance to degradation [1,2]. Microplastics (<5 mm), derived from both primary sources and the fragmentation of larger plastic debris, are readily ingested by aquatic organisms and can adsorb a wide range of co-occurring contaminants, thereby enhancing their bioavailability and inducing multiple adverse effects, including oxidative stress, metabolic disturbance and immune dysfunction [3,4,5].

In addition to direct physiological impacts, MP can disrupt gut microbiota, which plays a central role in host metabolism, immune regulation, and environmental adaptation [6,7,8]. MP-induced microbial dysbiosis, characterized by reduced diversity and shifts in dominant taxa, has been widely reported in aquatic organisms, often involving enrichment or depletion of specific bacterial groups [9,10,11]. Such dysbiosis differs from symbiosis, which represents a stable host–microbiota relationship, and from probiotic or prebiotic interventions that aim to restore microbial balance [7,8]. These microbiota alterations may contribute to host physiological dysfunction, including impaired digestion, immune imbalance, and increased susceptibility to environmental stress [12,13]. However, a critical unresolved question remains: do MP-induced microbiota shifts exacerbate host toxicity, or do they represent adaptive responses that contribute to stress tolerance? Furthermore, the functional roles of specific microbial taxa enriched under MP exposure remain largely unclear.

The brine shrimp *Artemia salina* provides a powerful model to address these questions due to its ecological relevance, sensitivity to environmental stressors, and non-selective filter-feeding behavior, leading to the ingestion, accumulation, and retention of microplastics in the digestive tract [14,15,16]. Its widespread use in aquaculture raises concerns regarding trophic transfer and ecosystem-level effects [17,18,19]. These features enable the integration of physiological and microbiological analyses to dissect host–microbe interactions under environmental stress.

In this study, we investigated the chronic effects of polystyrene microplastics (PS-MPs), a widely prevalent and well-characterized model microplastic, on *A. salina* by integrating analyses of growth performance, physiological responses, and gut microbiota dynamics. More importantly, we aimed to determine whether microbiota alterations are functionally involved in mediating host responses to microplastic exposure, rather than being merely correlative. By combining microbial profiling with bacterial supplementation experiments, this study provides new insight into the role of gut microbiota in shaping host resilience under environmental stress.

## 2. Materials and Methods

### 2.1. Experimental Design and Materials

All experiments were performed under controlled laboratory conditions to ensure reproducibility and minimize environmental variability. This study aimed to investigate the physiological and microbiota responses of *A. salina* to polystyrene microplastics (PS-MPs) exposure under standardized environmental conditions.

*A. salina* cysts were obtained from Weifang Haohai Aquatic Products Co., Ltd. (Weifang, China) and stored at −20 °C. Artificial seawater was prepared using crude sea salt obtained from the Weifang Binhai Saltworks (Weifang, China) dissolved in distilled water. Hatching and culture were conducted at 25 ± 1 °C, salinity 28 ± 1‰, pH 8.1 ± 0.5, under a 12 h light/12 h dark photoperiod following standard *Artemia* culture protocols [20]. Salinity was maintained at a constant level throughout the experiment to ensure reproducibility and to avoid confounding effects. PS-MPs were purchased from Tianjin Junyijia Co., Ltd. (Tianjin, China).

### 2.2. Artemia Culture and PS-MPs Exposure

*Artemia* cysts were hatched in aerated artificial seawater (28‰ salinity) at a density of 0.2 g/L. Six groups were established, including a control (CK) and five PS-MPs exposure groups (1, 25, 50, 75, and 100 mg/L), each with three biological replicates [10]. PS-MPs suspensions were prepared gravimetrically and homogenized prior to use, as described previously [18]. *Artemia* were maintained at 25 ± 1 °C under a 12 h light/12 h dark photoperiod and fed daily with *Dunaliella salina* and yeast. The exposure medium was renewed every 48 h.

### 2.3. Bioaccumulation Assessment

*Artemia* exposed to 75 mg/L PS-MPs for 15 days were rinsed, fixed in 4% paraformaldehyde (Solarbio, Beijing, China), and examined using an optical microscope (Olympus, Tokyo, Japan) to assess microplastic accumulation. 

### 2.4. Survival and Growth Measurement

Survival was monitored daily for 15 days. Dead individuals were identified by the absence of movement after stimulation. Growth was assessed by measuring body length using an optical microscope (Olympus, Tokyo, Japan) with cellSens software version 4.5 (Olympus, Tokyo, Japan). 

### 2.5. Enzyme Activity Assays

*Artemia* samples were homogenized in Tris-HCl buffer (Solarbio, Beijing, China) and centrifuged. Digestive enzyme activities (protease, amylase, lipase, cellulase) were measured using standard methods, and Phenoloxidase (PO) activity was measured using L-DOPA as substrate [21].

Antioxidant enzyme activities, including catalase (CAT) and superoxide dismutase (SOD), were determined using commercial assay kits (Solarbio, Beijing, China) according to the manufacturer’s instructions.

### 2.6. Bacterial Isolation and Functional Validation

Gut bacteria were isolated, cultured, and identified by 16S rRNA sequencing following standard microbiological protocols [10]. Selected strains were used for functional validation experiments.

### 2.7. Microbiome Sequencing and Analysis

DNA extraction, PCR amplification, and sequencing were performed as previously described [10]. Data were processed using QIIME 2 version 2023.5, and taxonomic assignment was conducted against the SILVA SSU database version 138.

### 2.8. Microbial Diversity and Community Analysis

Alpha and beta diversity analyses were conducted using QIIME 2 version 2023.5 and R software version 4.3.1 (R Foundation for Statistical Computing, Vienna, Austria). PERMANOVA analysis was performed using the vegan package version 2.6-4 in R.

### 2.9. Statistical Analysis

All data are presented as mean ± standard deviation (SD). Statistical analyses were performed using GraphPad Prism 10 (GraphPad Software, San Diego, CA, USA). Differences were analyzed using Student’s *t*-test or ANOVA as appropriate. A value of *p* < 0.05 was considered statistically significant.

## 3. Results

### 3.1. PS-MPs Impair Survival, Growth, and Induce Phenotypic Alterations in A. salina

**Figure 1:**
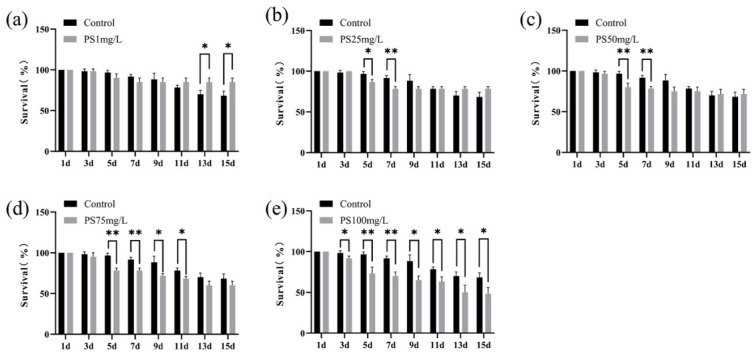
Survival of *A. salina* under different concentrations of PS-MPs during the exposure period. (**a**–**c**) Survival curves for control and low-concentration groups (≤50 mg/L). (**d**,**e**) Survival curves for 75 and 100 mg/L groups. Data are presented as mean ± SD (*n* = 3 biological replicates). Statistical significance was determined using two-way ANOVA with Tukey’s multiple comparisons test. * *p* < 0.05, ** *p* < 0.01.

To systematically evaluate the impact of PS-MPs on organismal performance, survival and growth were monitored across a gradient of exposure concentrations. PS-MPs exposure resulted in clear concentration-dependent effects. No significant differences were observed between control and low-dose groups (1–50 mg/L), whereas higher concentrations (75 and 100 mg/L) led to a significant decline in survival over time (Figure 1). Mortality remained relatively low during the early phase but increased markedly during the mid-to-late exposure period, suggesting cumulative toxicity.

**Figure 2:**
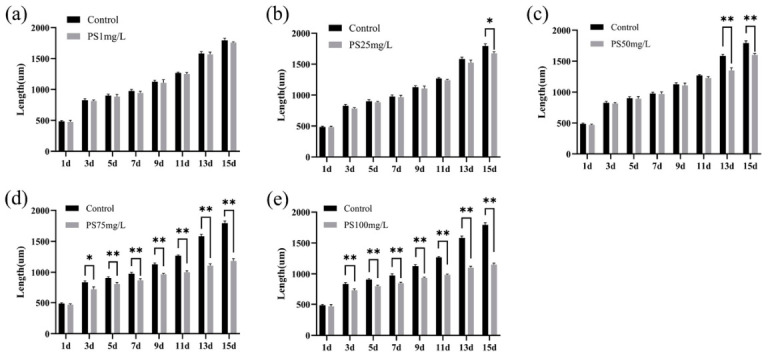
Body length of *A. salina* under different concentrations of PS-MPs during the exposure period. (**a**–**c**) Body length at PS-MPs concentrations of 1, 25, and 50 mg/L. (**d**,**e**) Body length at 75 and 100 mg/L. Data are presented as mean ± SD (*n* = 3 biological replicates). Statistical significance was determined using two-way ANOVA with Tukey’s multiple comparisons test. * *p* < 0.05, ** *p* < 0.01.

Growth exhibited a similar dose-dependent pattern. Body length remained comparable to controls at lower concentrations but was significantly reduced at ≥50 mg/L, with earlier onset and greater magnitude of inhibition observed at higher exposure levels (Figure 2). In addition to quantitative changes, PS-MPs exposure induced noticeable phenotypic alterations, including reduced motility, decreased feeding activity, and smaller body size, particularly in the high-concentration groups, indicating impaired physiological performance.

**Figure 3:**
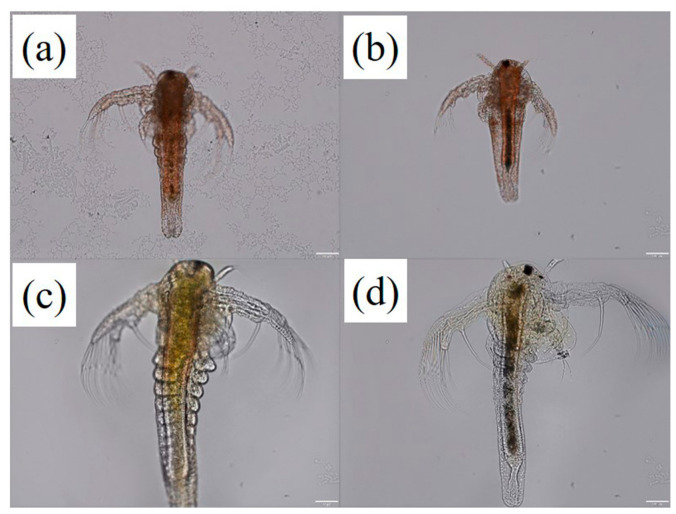
Intestinal accumulation of PS-MPs in *A. salina*. Representative images showing gut morphology and particle accumulation in larval (**a**,**b**) and adult (**c**,**d**) *A. salina*. (**a**,**c**) Control groups; (**b**,**d**) PS-MPs exposure (75 mg/L). Scale bars = 100 μm.

Given that 75 mg/L significantly reduced survival and growth and represented the lowest concentration that consistently induced measurable effects without excessive mortality, it was selected for subsequent sublethal mechanistic analyses. Microscopic examination revealed pronounced accumulation of PS-MPs within the intestinal tract, with dense particle aggregates clearly visible in exposed individuals, whereas no accumulation was observed in control groups (Figure 3).

### 3.2. PS-MPs Disrupt Digestive, Immune, and Antioxidant Functions

**Figure 4:**
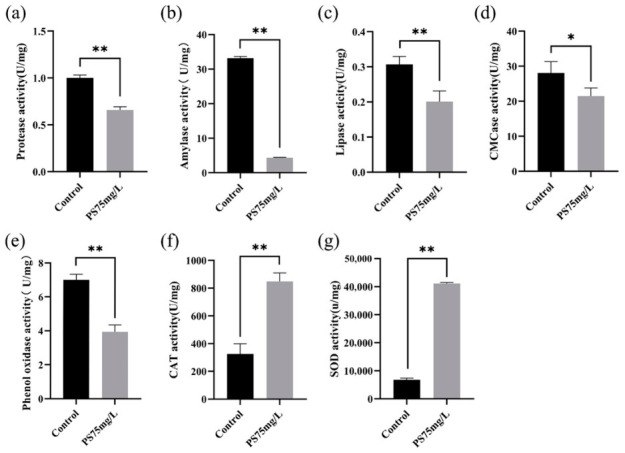
Effects of PS-MPs on digestive, immune-related, and antioxidant enzyme activities in *A. salina*. (**a**–**d**) Digestive enzyme activities, including protease, amylase, lipase, and cellulase. (**e**) phenoloxidase (PO) activity; (**f**,**g**) antioxidant enzyme activities, including catalase (CAT) and superoxide dismutase (SOD). All measurements were performed after 15 days of exposure to 75 mg/L PS-MPs. Data are presented as mean ± SD (*n* = 3 biological replicates). Statistical significance was determined by Student’s *t*-test. * *p* < 0.05, ** *p* < 0.01.

To further assess physiological responses to PS-MPs exposure, key digestive, immune-related, and antioxidant enzyme activities were measured. Exposure resulted in a consistent suppression of digestive capacity, as evidenced by significant reductions in protease, amylase, lipase, and cellulase activities compared with controls (Figure 4a–d). Among these, amylase exhibited the most pronounced decrease. Phenoloxidase (PO) activity was also significantly reduced (Figure 4e), indicating an overall decline in immune-related function.

In contrast, antioxidant enzyme activities, including catalase (CAT) and superoxide dismutase (SOD), were significantly increased following exposure (Figure 4f,g). These results reveal a coordinated physiological response characterized by reduced digestive and immune activities alongside enhanced antioxidant enzyme activity.

### 3.3. PS-MPs Reduce Gut Microbial Richness and Diversity

**Figure 5:**
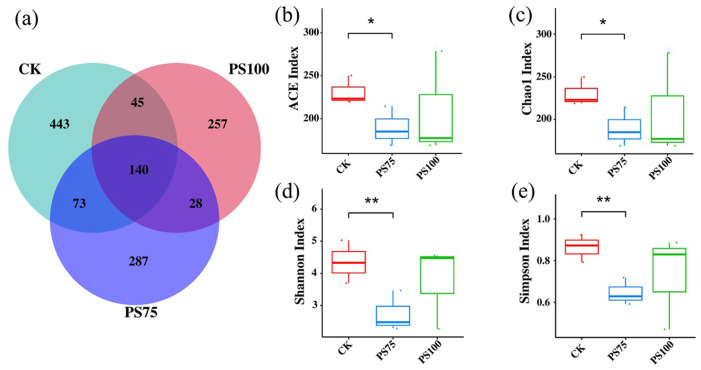
Effects of PS-MPs exposure on gut microbial richness and diversity in *A. salina*. (**a**) Venn diagram showing shared and unique OTUs among different groups. (**b**–**e**) α-diversity indices, including ACE (**b**), Chao1 (**c**), Shannon (**d**), and Simpson (**e**). Data are presented as mean ± SD (*n* = 3 biological replicates). Statistical differences were analyzed using one-way ANOVA followed by appropriate post hoc tests. * *p* < 0.05, ** *p* < 0.01. Different colors represent different experimental groups.

To assess the effects of PS-MPs on gut microbial communities, both richness and diversity indices were analyzed. Microplastic exposure led to a clear reduction in microbial richness, as indicated by significant decreases in ACE and Chao1 indices compared with controls (Figure 5b,c).

Consistently, microbial diversity was also reduced, with both Shannon and Simpson indices showing significant declines, particularly at 75 mg/L (Figure 5d,e). The OTU-based Venn diagram further revealed differences in shared and unique taxa among groups (Figure 5a). These results indicate that PS-MPs exposure alters gut microbial community structure, characterized by reduced richness and diversity.

### 3.4. PS-MPs Reshape Gut Microbial Community Composition

**Figure 6:**
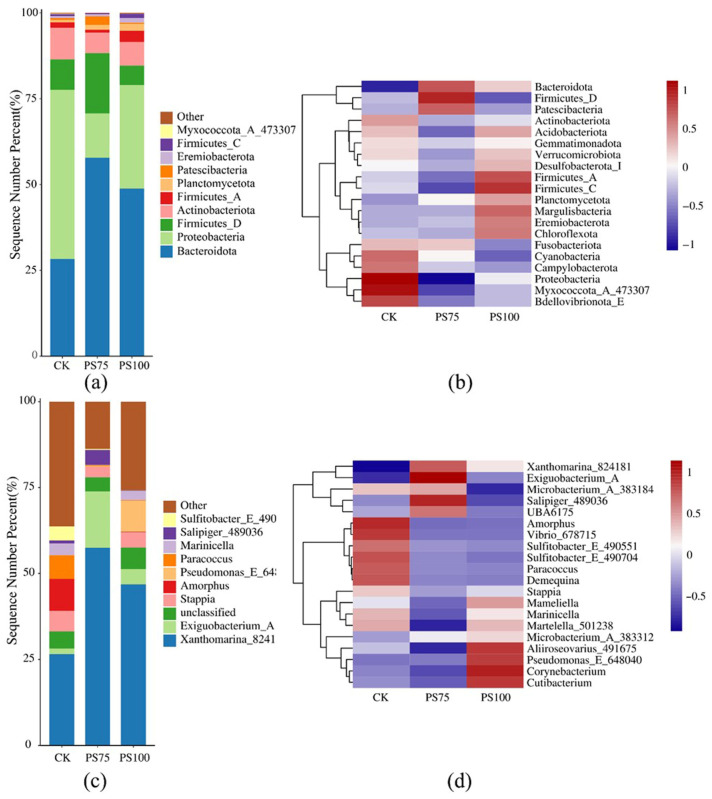
Effects of PS-MPs exposure on gut microbial community composition in *A. salina*. (**a**) Relative abundance of bacterial taxa at the phylum level. (**b**) Heatmap showing phylum-level composition across different groups. (**c**) Relative abundance of bacterial taxa at the genus level. (**d**) Heatmap showing genus-level composition across different groups. Data represent the relative abundance of microbial taxa in each group.

Consistent with the observed reduction in microbial diversity, PS-MPs exposure induced marked changes in gut microbial community composition. At the phylum level, control samples were dominated by Proteobacteria, whereas exposed groups showed an relative increase in Bacteroidota and Firmicutes, accompanied by a decline in Proteobacteria (Figure 6).

At the genus level, compositional shifts were more pronounced. Taxa such as *Pseudomonas* and *Exiguobacterium* were enriched following exposure, whereas others, including *Vibrio* and *Paracoccus*, were consistently reduced. These shifts were more evident at higher concentrations, indicating dose-dependent restructuring of the microbial community. These results demonstrate that PS-MPs exposure not only reduces diversity but also selectively reshapes microbial composition through coordinated enrichment and depletion of specific taxa.

### 3.5. Isolation and Identification of Culturable Gut Bacteria

To identify functionally relevant members of the gut microbiota, culturable bacteria were isolated and characterized. A total of 13 bacterial strains were isolated and identified by 16S rRNA sequencing (Table 1), representing multiple bacterial genera, including *Pseudomonas*, *Exiguobacterium*, and other taxa.

Notably, several isolates corresponded to taxa enriched in the microbiome analysis. Based on these enrichment patterns, *Pseudomonas knackmussii* and *Exiguobacterium profundum* were selected as representative strains for subsequent functional validation.

### 3.6. Functional Effects of Representative Bacterial Strains

**Figure 7:**
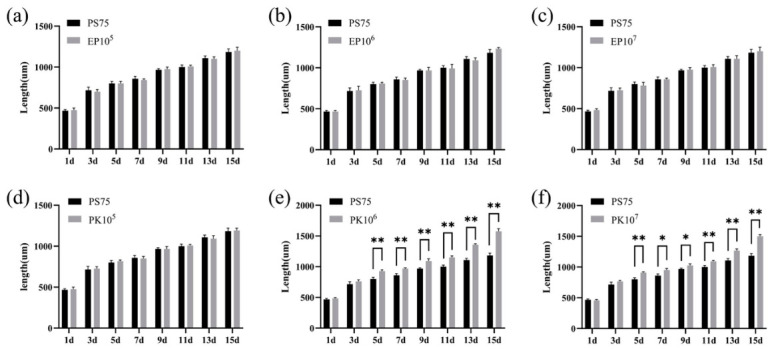
Effects of bacterial supplementation on growth of *A. salina* under PS-MPs exposure. (**a**–**c**) Body length following supplementation with *E. profundum* at different concentrations (1.45 × 10^5^–10^7^ CFU/mL); (**d**–**f**) body length following supplementation with *P. knackmussii* (1.2 × 10^5^–10^7^ CFU/mL). Data are presented as mean ± SD. Statistical significance was determined using two-way ANOVA with Tukey’s multiple comparisons test. * *p* < 0.05, ** *p* < 0.01.

To evaluate whether specific taxa contribute to host responses under PS-MPs exposure, supplementation experiments were performed under a fixed PS-MPs concentration (75 mg/L) with different bacterial doses. Supplementation with *E. profundum* had no detectable effect on host growth across the tested concentrations (Figure 7a–c). In contrast, *P. knackmussii* significantly promoted growth in a concentration-dependent manner, with the most pronounced effect observed at intermediate concentrations (Figure 7d,e).

**Figure 8:**
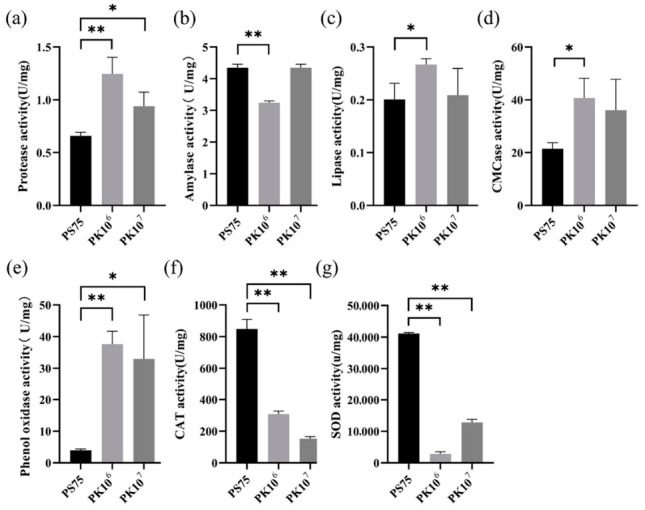
Enzymatic responses of *A. salina* following *P. knackmussii* supplementation under PS-MPs exposure. (**a**–**d**) Digestive enzyme activities (protease, amylase, lipase, and cellulase); (**e**) phenoloxidase (PO) activity; (**f**–**g**) antioxidant enzyme activities (CAT and SOD). Data are presented as mean ± SD. Statistical significance was determined using two-way ANOVA with Tukey’s multiple comparisons test. * *p* < 0.05, ** *p* < 0.01.

Further analysis showed that *P. knackmussii* supplementation partially restored digestive enzyme activities, including protease, amylase, lipase, and cellulase, and increased phenoloxidase activity. In parallel, antioxidant enzyme activities (CAT and SOD) were reduced compared with the PS-MPs-exposed group (Figure 8). These results demonstrate that *P. knackmussii* alleviates PS-MPs-induced physiological impairment.

## 4. Discussion

In this study, we investigated the effects of PS-MPs on host physiology and gut microbiota in *A. salina*. Our results demonstrate that PS-MPs exposure impairs growth and survival, disrupts digestive and immune functions, and induces oxidative stress. In parallel, PS-MPs exposure alters gut microbial diversity and composition, and specific bacterial taxa can modulate host responses under stress conditions. These findings highlight the interconnected roles of physiological and microbiota-mediated responses in shaping host adaptation to environmental stressors.

The observed physiological impairments are consistent with previous studies showing that microplastics negatively affect growth, metabolism, and immune function in aquatic organisms [4,22,23]. For example, reduced feeding efficiency, inhibited growth, and increased oxidative stress have been reported in fish, crustaceans, and zooplankton exposed to microplastics [24,25,26]. The reduction in digestive enzyme activities observed in this study is in line with these reports and suggests that microplastics may interfere with nutrient assimilation and digestive processes. Similarly, the increase in antioxidant enzyme activities, such as CAT and SOD, reflects a typical stress response to environmental pollutants, indicating the activation of defense mechanisms against oxidative damage [27,28].

In addition to physiological effects, our results demonstrate that PS-MPs exposure leads to significant alterations in gut microbial communities. Reduced microbial richness and diversity, together with compositional shifts, have been widely reported in previous studies of microplastic exposure [29,30]. These changes are often associated with microbial dysbiosis, which may compromise host health by disrupting metabolic and immune-related functions [31,32]. Our findings are consistent with these observations and further reveal that specific taxa respond differently to PS-MPs exposure, with some groups being enriched while others are depleted. However, a non-monotonic pattern was observed, with 75 mg/L exerting a stronger suppressive effect on microbial diversity than 100 mg/L. This may reflect differential responses to stress intensity, where moderate exposure disrupts community stability, while higher concentrations impose stronger selection for stress-tolerant taxa, partially stabilizing diversity metrics.

Notably, the enrichment of taxa such as *Pseudomonas* and *Exiguobacterium* suggests that certain bacteria may be more adaptable to microplastic-associated environments. Members of the genus *Pseudomonas* are known for their metabolic versatility and ability to tolerating environmental stressors [29,33], which may explain their increased abundance under PS-MPs exposure. In contrast, the reduction in taxa such as *Vibrio* and *Paracoccus* may reflect their sensitivity to altered gut conditions or competition with more stress-tolerant species. These compositional shifts indicate that PS-MPs exposure not only reduces diversity but also selectively reshapes the microbial community.

A key finding of this study is that specific microbial taxa can actively influence host responses to microplastic stress. In particular, *P. knackmussii* significantly promoted host growth and partially restored physiological functions, whereas *E. profundum* showed no detectable effect. This functional divergence highlights that changes in microbiota composition are not merely descriptive but can have direct biological consequences for the host. The ability of *P. knackmussii* to alleviate physiological impairment may be associated with its metabolic versatility, including enhanced nutrient processing, production of bioactive metabolites, and potential modulation of host oxidative stress and immune responses [34,35]. Such functions may improve energy availability and contribute to maintaining physiological homeostasis under environmental stress conditions. Whether these beneficial effects arise from direct metabolic contributions of *P. knackmussii* or from its ability to reshape the overall gut microbiota toward a more resilient community remains unclear. These two mechanisms are not mutually exclusive and may act in concert, warranting further investigation.

These results support the idea that gut microbiota play an active role in mediating host responses to environmental stressors. Notably, the beneficial effects of *P. knackmussii* indicate that microplastic-induced shifts in gut microbiota are not solely detrimental but may also represent adaptive responses that enhance host tolerance. This finding highlights the capacity of microbiota to buffer pollutant-induced stress and underscores the ecological significance of host–microbe interactions in shaping organismal resilience. Accordingly, microbial communities should be viewed not as passive indicators of environmental change, but as active contributors to host adaptation, consistent with emerging evidence in ecological and toxicological studies [36,37].

Despite these findings, several limitations should be considered. First, although physiological and microbiota responses were characterized, histological analysis was not performed to assess tissue-level alterations. Second, the concentrations of microplastics used in this study are higher than those typically reported in natural environments, which may limit direct ecological extrapolation. Third, the underlying mechanisms by which specific bacterial taxa modulate host responses remain unclear. Future studies integrating histological approaches, metabolomic analyses, and environmentally relevant exposure conditions will provide a more comprehensive understanding of these processes.

Overall, our study demonstrates that PS-MPs exposure disrupts host physiology and reshapes gut microbiota, and that specific bacterial taxa can modulate host responses under stress conditions. These findings provide new insight into the functional role of microbiota in mediating host resilience to environmental pollutants and highlight the importance of considering host–microbe interactions in assessing the biological impacts of microplastics.

## 5. Conclusions

In this study, we demonstrate that polystyrene microplastics (PS-MPs) impair growth, survival, and physiological functions in *A. salina*, accompanied by oxidative stress and disruption of digestive and immune processes. In parallel, PS-MPs exposure significantly alters gut microbial diversity and community composition.

Future studies should aim to elucidate the causal mechanisms underlying microbiota-mediated host responses using gnotobiotic systems and multi-omics approaches. In addition, investigating environmentally relevant microplastic mixtures and long-term exposure scenarios will be essential for improving ecological relevance.

From an applied perspective, our results suggest that targeted manipulation of gut microbiota may represent a potential strategy to mitigate microplastic-induced toxicity in aquatic organisms.

## Figures and Tables

**Table 1:** Culturable gut bacterial isolates from *A. salina* following 15 days of PS-MPs exposure.

Isolate ID	DNA Identification	Sequence Homology (%)
PS1_1	*Pseudomonas knackmussii*	98.95%
PS1_3	*Microbacterium algeriense*	99.79%
PS1_5	*Microbacterium oxydans*	99.44%
PS2_1	*Rossellomorea arthrocnemi*	99.31%
PS2_2	*Rossellomorea aquimaris*	99.17%
PS2_3	*Microbacterium esteraromaticum*	99.29%
PS2_5	*Microbacterium aurantiacum*	99.79%
PS3_1	*Ectopseudomonas chengduensis*	99.58%
PS4_1	*Stutzerimonas stutzeri*	99.65%
PS4_3	*Chryseobacterium takakiae*	98.43%
PS5_2	*Exiguobacterium profundum*	99.72%
PS5_3	*Glutamicibacter protophormiae*	99.15%
PS5_5	*Bacillus haikouensis*	99.31%

## Data Availability

The original contributions presented in this study are included in the article. Further inquiries can be directed to the corresponding author.

## Abbreviations

The following abbreviations are used in this manuscript:

MP/MPs	Microplastic(s)
PS-MPs	Polystyrene microplastics
SOD	Superoxide dismutase
CAT	Catalase
PO	Phenoloxidase
OTUs	Operational taxonomic units
ASVs	Amplicon sequence variants
PCoA	Principal coordinates analysis
PERMANOVA	Permutational multivariate analysis of variance
CFU	Colony-forming units
CK	Control group
DNS	3,5-dinitrosalicylic acid
p-NPP	p-nitrophenyl palmitate
CMC	Carboxymethyl cellulose
